# Genetic Regulation of Physical Fitness in Children: A Twin Study of 15 Tests from Eurofit and Fitnessgram Test Batteries

**DOI:** 10.1249/MSS.0000000000003496

**Published:** 2024-07-01

**Authors:** KARRI SILVENTOINEN, JOSÉ MAIA, ELINA SILLANPÄÄ, REIJO SUND, ÉLVIO R. GOUVEIA, ANTÓNIO ANTUNES, GONÇALO MARQUES, MARTINE THOMIS, JAAKKO KAPRIO, DUARTE FREITAS

**Affiliations:** 1 Population Research Unit, Faculty of Social Sciences, University of Helsinki, Helsinki, FINLAND; 2 Research Institute of Human Development, Kyoto International Social Welfare Exchange Centre, Kyoto, JAPAN; 3 Centre of Research, Education, Innovation and Intervention in Sport (CIFI2D), Faculty of Sport, University of Porto, Porto, PORTUGAL; 4 Gerontology Research Center, Faculty of Sport and Health Sciences, University of Jyväskylä, Jyväskylä, FINLAND; 5 The Wellbeing Services County of Central Finland, Jyväskylä, FINLAND; 6 Institute of Clinical Medicine, University of Eastern Finland, Kuopio, FINLAND; 7 Department of Physical Education and Sport, University of Madeira, Funchal, PORTUGAL; 8 LARSYS, Interactive Technologies Institute, Funchal, PORTUGAL; 9 Physical Activity, Sports & Health Research Group, Department of Movement Sciences, Faculty of Movement and Rehabilitation Sciences, KU Leuven, Leuven, BELGIUM; 10 Institute for Molecular Medicine Finland (FIMM), HiLIFE, University of Helsinki, Helsinki, FINLAND

**Keywords:** CHILDREN, GENETIC CORRELATIONS, HERITABILITY, PHYSICAL FITNESS

## Abstract

**Purpose:**

This study aimed to analyze the shared genetic background of physical fitness tests in children.

**Methods:**

Physical fitness was assessed in 198 Portuguese twin pairs (6–18 yr old, 40% monozygotic) through 15 tests from the Eurofit and Fitnessgram test batteries. Genetic twin modeling was used to estimate the heritability of each test and the genetic correlations between them.

**Results:**

Girls performed better than boys in flexibility, whereas boys performed better than girls in cardiorespiratory endurance and muscular strength. No sex differences were found in the influence of genetic factors on the physical fitness tests or their mutual correlations. Genetic factors explained 52% (standing long jump) to 79% (sit and reach) of the individual variation in motor performance, whereas individual-specific environmental factors explained the remaining variation. Most of the tests showed modest to moderate genetic correlations. Out of all 105 genetic correlations, 65% ranged from 0.2 to 0.6 indicating that they shared from 4% to 36% of genetic variation. The correlations between individual-specific environmental factors were mostly negligible.

**Conclusions:**

Tests measuring the strength of different muscle groups showed only modest correlations, but moderate correlations were found between tests measuring explosive strength, running speed/agility, and cardiorespiratory endurance. Genetic factors explained a major portion of the variation in tests included in the Eurofit and Fitnessgram test batteries and explained the correlations between them. The modest to moderate genetic correlations indicated that there is little redundancy of tests in either Eurofit or Fitnessgram test batteries.

High levels of physical fitness in childhood play a key role in a healthy adult life (1). However, physical fitness is not a single operational concept but rather a combination of interrelated components, most importantly cardiorespiratory endurance, muscular strength, and agility (2). Each component of physical fitness might be related to somewhat different health outcomes, even when they all predict good health (3). During the last decades, the levels of physical fitness have declined in school-aged children and adolescents creating a major public health challenge (4,5). Physical fitness assessment is crucial in responding to this challenge because it allows us to evaluate the need and effectiveness of intervention programs and to identify and follow children who need additional support. In the optimal case, the tests used to assess physical fitness should be valid, reliable, and practical so that they can be conducted in schools and youth sports settings without requiring specialized instruments or technical expertise (6).

Studying the factors behind individual differences in cardiorespiratory endurance, muscular strength, and motor performance can also provide insight into the background of these physical fitness components. There is a strong body of evidence on the importance of genetic factors behind the variation in physical fitness. Twin studies have shown moderate heritability for different measures of physical fitness (7–9), and genome-wide association studies have identified a number of genetic variants associated with muscular strength (10) and cardiorespiratory endurance (11). There is also information on how genetic factors contribute to the covariation between physical fitness tests. A Dutch twin study with information on four physical fitness tests (7) and a Swedish twin and family study with information on three muscular strength tests (12) found that the major part (47%–99%) of the covariation between these tests was due to shared genetic factors. A limitation of these studies is the small number of tests, which does not allow for a full assessment of the components of physical fitness.

A comprehensive analysis of the shared genetic background of multiple tests covering different components of physical fitness would have both scientific and clinical importance. From a scientific perspective, a high genetic correlation between two tests may suggest a shared biological background, such as measuring the performance of the same muscle groups. From a clinical perspective, high correlations indicate redundancy and can help select the most informative tests, especially if the time for testing is limited. This study addresses this gap using twin data on children who performed 15 tests from two widely used physical fitness test batteries. We aim to analyze the underlying structure of correlations between all tests and to estimate how much they share common genetic variation. The twin design also allows for controlling intraindividual variation in physical performance, which can contribute to the correlations of performance in different physical fitness tests.

## DATA AND METHODS

Our study cohort was derived from the Madeira Twin Family Study (13). Information on twins was obtained by contacting the executive boards of all public and private schools in the Autonomous Region of Madeira, Portugal, and asking whether they had twin students. Invitation letters were then sent to 434 families with twins. A total of 216 families participated in a clinical exam in the capital city of Funchal. We removed 18 twin pairs less than 6 yr of age because the physical fitness tests were not adjusted to these ages, and thus, we had 198 twin pairs (6–18 yr of age; 51% girls) in the analyses. The Scientific Board of the University of Madeira approved the study protocol. The participants and/or their parents or legal guardians provided written informed consent. Zygosity was assessed based on 15 autosomal genetic markers and a sex-determining marker (AmpFISTR Identifiler kit) from a blood test the children gave during the clinical examination (14): 78 were monozygotic (MZ), 69 same-sex dizygotic, and 51 opposite-sex dizygotic pairs.

Physical fitness was assessed through two test batteries: the Eurofit and the Fitnessgram. The Eurofit included nine tests (flamingo balance, plate tapping, sit and reach, standing long jump, handgrip, sit-ups, bent arm hang, shuttle run of 10 × 5 m, and 12-min run/walk). The Fitnessgram included six tests (sit and reach right, sit and reach left, trunk lift, curl up, push-up, and 20-m shuttle run). Researchers with extensive experience in Kinanthropometry assessed the participants according to the Eurofit (15) and Fitnessgram (16) test battery protocols. The children performed the tests with their twin siblings and were accompanied by their parents. In statistical modeling, we reversed the scales of flamingo balance, plate tapping, and shuttle run of 10 × 5 m so that for all tests higher values indicate better performances. Furthermore, we adjusted the physical fitness tests for age and the square of age because they showed statistically significant associations with most of them. This was done by calculating regression residuals for each test separately in boys and girls using the Stata statistical package, version 17 for Windows (StataCorp, College Station, TX). Because means and SD differed between boys and girls (Supplemental Table 1, Supplemental Digital Content, http://links.lww.com/MSS/D54) for most tests, we also standardized them in boys and girls to be able to present pooled analyses. However, we presented the sex-specific results in appendixes to confirm that presenting pooled analyses does not conceal any differences between boys and girls. Linear regression models were used for statistical testing in descriptive analyses after correcting the standard errors for the lack of statistical independence of twins sampled as pairs (17).

We used genetic twin modeling to obtain information on the contribution of genetic and environmental factors to the variation and covariation of the test items (18). This method is based on the principle that MZ twins share virtually the same genomic sequence, whereas dizygotic (DZ) twins share, on average, 50% of their genes identical-by-descent, similar to ordinary siblings. This allows for estimating the underlying correlation structure within co-twins and decomposing the variation of the performance in each test and the covariation between the performance in different tests into genetic and environmental components. Additive genetic variance (A; correlation 1 within MZ and 0.5 within DZ pairs) includes the effects of all loci affecting the test performance. Shared environmental variance (C; correlation 1 within both MZ and DZ pairs) includes the effects of all environmental factors that make co-twins similar. Unique environmental variance (E; correlation 0 within both MZ and DZ twins) includes the effects of all environmental factors that make co-twins different including measurement error and interindividual fluctuation in physical performance.

We started the analyses using univariate genetic models to test the assumptions of twin modeling, find the best-fitting model, and calculate heritability estimates (18). DZ correlations were more than half of MZ correlations indicating a possible role of shared environmental factors (Supplemental Table 2, Supplemental Digital Content, http://links.lww.com/MSS/D54). Therefore, we used the additive genetic/shared environment/unique environment (ACE) model as the starting point of genetic analyses. The model fit statistics are presented in Supplemental Table 3 (Supplemental Digital Content, http://links.lww.com/MSS/D54). We did not find any evidence for sex-specific genetic effects and were able to have the same parameter estimates for boys and girls. When we applied a more parsimonious additive genetic/unique environment (AE) model, it fit well compared with the ACE model, suggesting that shared environmental factors are not needed in the model. This is also consistent with previous twin studies, as summarized in three meta-analyses, which did not find evidence of the effect of shared environmental factors on physical fitness (7–9). Thus, we used the AE model without sex-specific genetic factors and the same parameter estimates for boys and girls for further genetic modeling. For most physical fitness tests, this model fit the data well when compared with the saturated model—which does not make any assumptions and estimates all possible statistics freely—suggesting that there is no violation of the assumptions of twin modeling (i.e., the same means and SDs for both co-twins in the pair as well as MZ and DZ twins). Using the conventional *P* value of 0.05, less than optimal fit was found for 6 tests, but for three of them, the *P* value was above the Bonferroni-corrected *P* value (0.003 for 15 tests) and might be due to multiple testing. However, for bent arm hang, 12-min run/walk and curl up, the model suggested some violations. When we analyzed this in detail, we found some differences in means and SDs between MZ and DZ twins, but they did not show any systematic pattern (Supplemental Table 1, Supplemental Digital Content, http://links.lww.com/MSS/D54). Thus, we needed to expect that for these traits, twins are representative of the same basic population.

After conducting univariate models, we used bivariate Cholesky decomposition to calculate the genetic and environmental covariation underlying the correlations between the tests (19). Cholesky decomposition is a model-free method and thus does not make any assumption about the underlying correlation structure but decomposes all variation and covariation in the data into uncorrelated latent factors. Standardizing these covariates provides us with the estimates of additive genetic and unique environmental correlations. The genetic twin modeling was conducted using the OpenMx package, version 3.0.2, of the R statistical software (20). The model parameters and their 95% confidence intervals (CI) were estimated through the structural equation methodology and using the maximum likelihood estimator.

## RESULTS

Table 1 presents the descriptive statistics by sex. Girls were ahead of boys in all three sit and reach tests, whereas boys performed better than girls in the standing long jump, handgrip, sit-ups, bent arm hang, curl up, and push-up, as well as in the three running/walking tests. For flamingo balance, plate tapping, and trunk lift, there were no statistically significant differences between boys and girls.

**TABLE 1:** Means and SD of physical fitness tests by sex.

		Boys		Girls		*P* Value of Sex Difference*^a^*
		Mean	SD	Mean	SD	
Eurofit						
Flamingo balance (*N* of attempts)		21.0	8.27	21.4	8.01	0.7090
Plate tapping (s)		17.1	4.63	16.9	4.62	0.7720
Sit and reach (cm)		15.4	6.11	19.2	6.89	<0.0001
Standing long jump (cm)		130	33.1	116	25.5	<0.0001
Handgrip (kg)		19.7	10.58	17.1	8.22	0.0280
Sit-ups (*N* in 30 s)		16.9	6.91	14.0	5.85	<0.0001
Bent arm hang (s)		9.6	11.81	3.7	3.95	<0.0001
Shuttle run (10 × 5 m) (s)		23.4	3.39	24.6	2.57	0.0010
12-min run/walk (m)		1756	470	1524	366	<0.0001
Fitnessgram						
Sit and reach right (cm)		22.8	6.69	26.0	6.90	<0.0001
Sit and reach left (cm)		22.2	6.65	25.4	7.17	<0.0001
Trunk lift (cm)		24.8	5.28	25.7	4.78	0.1130
Curl up (*N*)		14.3	20.45	8.8	12.63	0.0070
Push-up (*N*)		8.5	7.97	5.0	4.93	<0.0001
20-m shuttle run (lap number)		27.8	18.0	19.0	10.6	<0.0001

*^a^* Adjusted for age.

We began the genetic modeling by using univariate genetic models to analyze the relative contributions of additive genetic and unique environmental factors to the physical fitness tests (Table 2). All tests showed moderate to high heritabilities. The proportions of individual variation explained by genetic factors varied from 52% (standing long jump) to 79% (sit and reach and bent arm hang). When the analyses were stratified by sex, no clear sex differences were observed. Boys showed higher heritability estimates than girls in some tests, whereas girls showed higher heritability estimates than boys in other tests (Supplemental Table 4, Supplemental Digital Content, http://links.lww.com/MSS/D54).

**TABLE 2:** Additive genetic and unique environmental variance components of physical fitness tests in boys and girls.

	Additive Genetic Factors			Unique Environmental Factors		
	*a* ^2^	95% CI		*e* ^2^	95% CI	
		LL	UL		LL	UL
Eurofit						
Flamingo balance	0.60	0.47	0.70	0.40	0.30	0.53
Plate tapping	0.72	0.61	0.79	0.28	0.21	0.39
Sit and reach	0.79	0.70	0.86	0.21	0.14	0.30
Standing long jump	0.52	0.37	0.64	0.48	0.36	0.63
Handgrip	0.71	0.61	0.78	0.29	0.22	0.39
Sit-ups	0.76	0.65	0.83	0.24	0.17	0.35
Bent arm hang	0.79	0.70	0.85	0.21	0.15	0.30
Shuttle run (10 × 5 m)	0.65	0.52	0.74	0.35	0.26	0.48
12-min run/walk	0.75	0.66	0.81	0.25	0.19	0.34
Fitnessgram						
Sit and reach right	0.74	0.63	0.81	0.26	0.19	0.37
Sit and reach left	0.74	0.65	0.81	0.26	0.19	0.35
Trunk lift	0.60	0.47	0.70	0.40	0.30	0.53
Curl up	0.62	0.48	0.72	0.38	0.28	0.52
Push-up	0.64	0.52	0.73	0.36	0.27	0.48
20-m shuttle run	0.76	0.68	0.83	0.24	0.17	0.32

LL and UL, the lower and upper limits of the CI.

**FIGURE 1:**
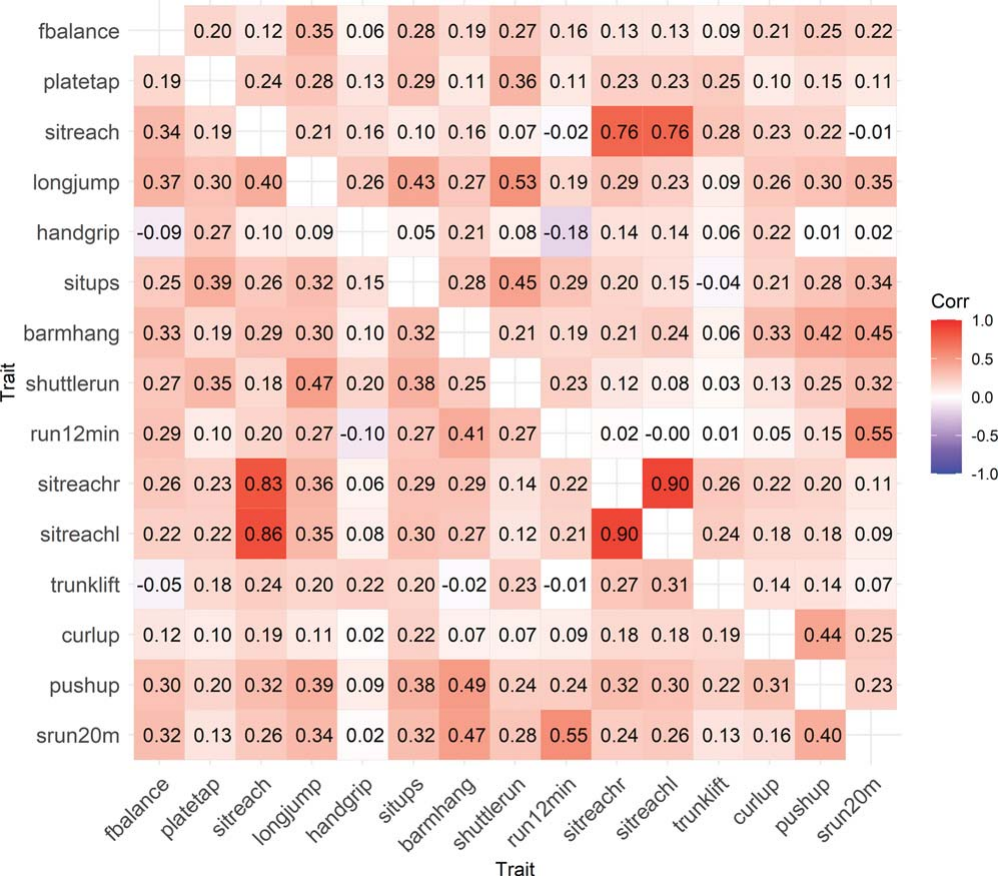
Trait correlations for boys (right triangular matrix) and girls (left triangular matrix) between physical fitness tests. Scales of flamingo balance, plate tapping, and shuttle run (10 × 5 m) were reversed, and therefore, a high score indicates a better performance. barmhang, bent arm hang; curlup, curl up; fbalance, flamingo balance; handgrip, handgrip; longjump, standing long jump; platetap, plate tapping; pushup, push-up; run12min, 12-min run/walk; shuttlerun, shuttle run 10 × 5 m; sitreach, sit and reach; sitreachl, sit and reach left; sitreachr, sit and reach right; situps, sit-ups; srun20m, 20-m shuttle run; trunklift, trunk lift.

We then analyzed the pairwise correlations between all physical fitness tests. Figure 1 presents the correlations between all tests in boys (right triangular matrix) and girls (left triangular matrix); the 95% CIs of these correlations are available in Supplemental Table 5 (Supplemental Digital Content, http://links.lww.com/MSS/D54). The trait correlations were roughly similar in boys and girls. The largest sex difference in these correlations was 0.27. For 70 of the 105 pairwise correlations, the difference was 0.10 or less. Only 12 of these correlations showed statistically significant sex differences (*P* < 0.05), and none of them were statistically significant using the Bonferroni-corrected *P* value (<0.00048 for 105 tests; Supplemental Table 6, Supplemental Digital Content, http://links.lww.com/MSS/D54).

**FIGURE 2:**
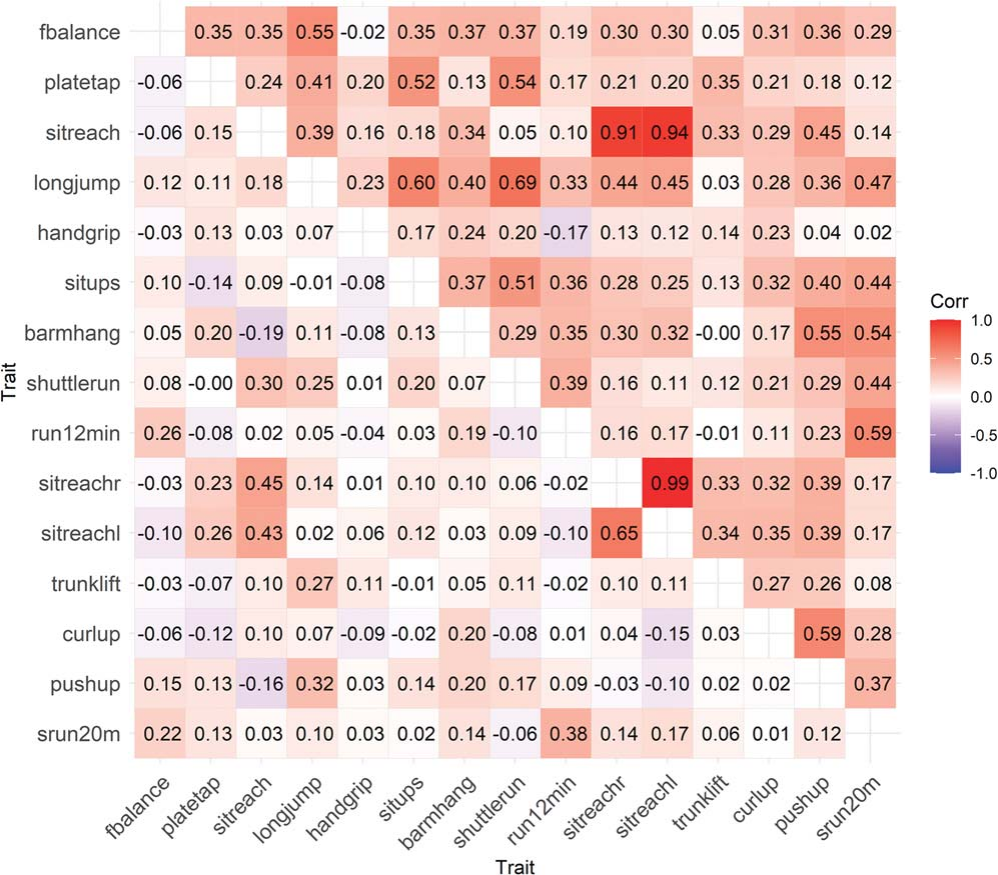
Additive genetic correlations (right triangular matrix) and unique environmental correlations (left triangular matrix) between physical fitness tests in boys and girls. Scales of flamingo balance, plate tapping, and shuttle run (10 × 5 m) were reversed, and therefore, a high score indicates a better performance. barmhang, bent arm hang; curlup, curl up; fbalance, flamingo balance; handgrip, handgrip; longjump, standing long jump; platetap, plate tapping; pushup, push-up; run 12min, 12-min run/walk; shuttlerun, shuttle run 10 × 5 m; sitreach, sit and reach; sitreachl, sit and reach left; sitreachr, sit and reach right; situps, sit-ups; srun20m, 20-m shuttle run; trunklift, trunk lift.

Finally, we conducted Cholesky decomposition to decompose these trait correlations into genetic and environmental components. Figure 2 presents additive genetic (right triangular matrix) and unique environmental correlations (left triangular matrix) in the pooled data of boys and girls; the 95% CIs of these correlations are available in Supplemental Table 7 (Supplemental Digital Content, http://links.lww.com/MSS/D54). Additive genetic correlations were systematically higher than the trait correlations (Fig. 1), whereas unique environmental correlations were weak and many of them negative. The highest genetic correlations (*r*_A_ ≥ 0.91) were found between the three sit and reach tests, but otherwise, most of them varied from modest to moderate. Generally, nearly all tests showed some genetic correlations with each other. Out of the 105 genetic correlations, 65% (68 genetic correlations) varied between 0.2 and 0.6 indicating that from 4% to 36% of the genetic variation was shared between these tests. When these analyses were stratified by sex, we did not find any systematic differences in the additive genetic (Supplemental Table 8, Supplemental Digital Content, http://links.lww.com/MSS/D54) or unique environmental correlations between boys and girls (Supplemental Table 9, Supplemental Digital Content, http://links.lww.com/MSS/D54).

## DISCUSSION

In this genetically informative study of children and adolescents, we aimed to gain more insight into the factors underlying individual variation in physical fitness. Boys outperformed girls in tests measuring cardiorespiratory endurance and muscular strength, as has been systematically demonstrated in previous studies (21). However, we found only minor sex differences in the genetic background of test performance. First, we did not find any evidence of sex-specific genetic factors, and the heritability estimates were roughly similar in both sexes. Second, we found that both trait and genetic correlations between physical fitness tests were roughly similar in boys and girls.

Our data allowed for the comparison of heritability estimates among several physical fitness tests, which has been rare in previous twin studies. Three meta-analyses reported the lowest heritability for balance (*a*^2^ = 0.35) and the highest heritability for V̇O_2max_ (*a*^2^ = 0.68) (7–9). However, the V̇O_2max_ was not objectively measured in the current study. A limitation of the previous meta-analysis is that these estimates come from different studies, making it difficult to compare them given the differences between the study populations. A notable exception is a Belgian study of 10-yr-old twins, which used the Eurofit test battery but included slightly different tests and protocols compared with our study (22). Because this study utilized a somewhat different statistical model, we recalculated the heritability estimates based on the reported twin correlations using the same model used in our study (the parameters’ estimates and modeling details are available in Supplemental Table 10, Supplemental Digital Content, http://links.lww.com/MSS/D54). The heritability estimates compared well with the ones of the current study. The sit-and-reach test showed the highest heritability in our study (*a*^2^ = 0.79) and in the Belgian study (*a*^2^ = 0.84), whereas the lowest heritability was found for the standing long jump (*a*^2^ = 0.52) and flamingo balance test (*a*^2^ = 0.48) in these studies, respectively.

When compared with other traits, the heritability estimates of physical fitness tests were similar or slightly lower than those for height (23) and body mass index (24) during childhood and adolescence and higher than those found for personality and other psychological traits in early adulthood (25). This suggests that physical fitness primarily reflects biological characteristics, whereas behavioral and psychological factors may also have an impact. The contributions of these different factors may also vary between test items, as seen in the varying heritability estimates. Interestingly, high heritability estimates for the sit-and-reach tests were found in the children who participated in our study and the 10-yr-old Belgian twin pairs (22). The high heritability of the sit-and-reach test may be due to its reflection of purely anatomical factors, whereas other tests, such as flamingo balance, may be affected by additional factors, such as training and personality, resulting in lower heritability. However, it is important to exercise caution when drawing any firm conclusions about the size of heritability estimates due to the mainly overlapping CIs.

Our results on the shared genetic background between the physical fitness tests are novel. Apart from the three sit-and-reach tests, which showed nearly perfect genetic correlation (*r*_A_ ≥ 0.91), the genetic correlations between the physical fitness tests varied from modest to moderate. In a Swedish study using clinical measures of knee extension, hand grip, and elbow flexion strength, moderate genetic correlations were found (*r*_A_ = 0.43–0.54), supporting the conclusion that different genetic components affect the performance of different muscle groups (12). It is worth noting that we found relatively high genetic correlations between tests measuring different components of physical fitness. For example, standing long jump showed a genetic correlation of 0.55 with flamingo balance, 0.60 with sit-ups, and 0.69 with shuttle run of 10 × 5 m. Nevertheless, even when moderate, the genetic correlations were much higher than the unique environmental correlations. This shows that genetic factors underlie the covariance of different physical fitness tests as found in previous studies (7,12). These genetic correlations also demonstrate that the correlations between these physical fitness tests are not caused by interindividual variation, as this variation is modeled as part of unique environmental correlations.

The multidimensional structure of physical fitness can contribute to the moderate or modest genetic correlations between the physical fitness tests. Physical fitness is influenced by both physiological factors, especially body composition (26), and behavioral factors, particularly physical activity (27,28), which may also be influenced by genetic factors (29,30). The genetic background of these physical fitness tests is likely multidimensional, including genetic factors associated with body composition, physical activity, and even motivation (31). Thus, it is understandable that, for example, the genetic predisposition to aerobic fitness develops lower limb strength and balance, leading to genetic correlations between them.

From a clinical perspective, the moderate and modest genetic correlations indicate little redundancy in the tests included in these test batteries. The exception is the two sit-and-reach tests from Fitnessgram, which seem unnecessary considering their very high trait and genetic correlations. The genetic correlations between trunk lift and the other tests were generally low, suggesting that this test captures variation not captured by the other tests. On the other hand, push-up, standing long jump, and 20-m shuttle run showed moderate correlations with nearly all other tests. Thus, if there is a possibility for only one or a few tests, these tests are good options because they capture variation from the whole test batteries. It is also interesting that many of the unique environmental correlations, even when small, were negative. This may suggest that the performance in one test negatively affects the performance in another test. Thus, extensive testing may decrease the reliability of individual tests.

There is previous evidence that both family- (32) and neighborhood-level factors can affect the physical fitness of children (33). However, all these effects are shared by co-twins and are thus modeled as part of shared environmental effects for which we did not find evidence. This is in accordance with a previous study from Madeira, which found little and unsystematic differences in physical fitness among children from high, average, and low socioeconomic status (34). These results suggest that there are no major differences between families and neighborhoods affecting physical fitness in our data. However, these results can be context-specific, and in some other populations, social-level factors can have more influence on the physical fitness in children.

Our data have both strengths and limitations. Our main strength is the very detailed measures of physical fitness—15 tests from two widely used physical fitness test batteries—in genetically informative data. To our knowledge, no previous study has been able to analyze in such detail the common genetic background of different physical fitness tests. In addition, Southern European populations are less well studied in human genetics than Northern European and North American populations of European ancestry. The children and adolescents who participated in the examination did so voluntarily, along with their twin siblings and parents. This may have increased their motivation compared with a scenario where the tests were conducted as part of physical education. Our main limitation is that the sample size was too small to study any age differences, and generally separating common environmental effects from additive genetic effects needs considerable statistical power (35). We did not find evidence that common environmental factors would affect the variation of physical performance. However, it is still possible that common environmental effects may be present in early childhood but disappear as children grow up and gain more independence, as found, for example, for body mass index (24). This would require larger samples and preferably longitudinal data to allow investigating how much genetic factors contribute to the correlations of physical fitness tests over age, as demonstrated previously (36). Twin studies including standardized interventions would also be important to analyze the role of genetic factors in the response to interventions. The extensive testing may also have negatively influenced the motor performance of the children. This does not affect genetic correlations but may have decreased unique environmental correlations and make some of them negative.

## CONCLUSIONS

In conclusion, genetic factors explain a major part of the variation in tests from the Eurofit and Fitnessgram test batteries and almost exclusively account for their mutual correlations. However, the genetic correlations between the tests mainly varied from modest to moderate, which may reflect the multidimensional structure of physical fitness. Because the genetic correlations are generally not high, there is not likely to be redundancy in either of the Eurofit and Fitnessgram test batteries. This is important because extensive testing can negatively affect the validity of individual tests.
